# Clustering and Switching in Semantic Verbal Fluency: Their Development and Relationship with Word Productivity in Typically Developing Greek-Speaking Children and Adolescents

**DOI:** 10.3390/jintelligence11110209

**Published:** 2023-11-01

**Authors:** Alexandra Karousou, Dimitra Economacou, Nikos Makris

**Affiliations:** 1 Department of Education Sciences in Early Childhood, Democritus University of Thrace, 681 00 Alexandroupolis, Greece; 2 Department of Primary Education, Democritus University of Thrace, 671 32 Xanthi, Greece; doikonom@eled.duth.gr (D.E.); nmakris@eled.duth.gr (N.M.)

**Keywords:** semantic verbal fluency, clustering, switching, number of clusters, number of switches, mean cluster size, maximum cluster size, typical development, children, adolescents, Greek language

## Abstract

Performance in semantic verbal fluency (SVF) tasks, mainly measured by the number of words of a particular semantic category produced within a limited time, is a widely accepted measure of cognitive functioning used in the neuropsychological assessment of children and adults. Two strategic processes, Clustering and Switching (C&S) have been proposed to underlie fluency processes and affect performance in the task. However, few studies have reported on the development of those cognitive strategies and their relationship with word productivity in typically developing children. Even fewer studies have covered a broad developmental period from preschool to adolescence or measured the effect of contextual factors in this relationship. Based on a sample of 472 typically developing Greek-speaking children aged 4;0 to 16;11 years, we investigated the development of SVF performance and reported on the degree to which it is affected by C&S strategies, children’s sex, and level of parental education. Results revealed a large effect of age on word productivity and on the use of C&S strategies. Two switching factors (number of clusters and number of switches) and two clustering factors (mean cluster size and a novel measure, maximum cluster size), appeared to be significantly associated with word productivity, with the largest effect being attributed to the two switching factors. C&S factors, together with children’s age and parental education, predicted 91.7% of the variance in the SVF score. Children’s sex was not found to have a significant effect on either word productivity or C&S strategies. Results are discussed for their theoretical implications on the strategic processes underlying word production in typically developing children.

## 1. Introduction

Verbal fluency tasks assess an individual’s ability to rapidly search, retrieve, and produce words stored in their mental lexicon. They typically require participants to produce as many words as possible from a given category (semantic verbal fluency, SVF) or words beginning with a specific letter or sound (phonemic verbal fluency, PVF) within a designated time frame, usually 60 s.

Despite their simplicity, these tasks tap into many cognitive processes, including lexico-semantic knowledge and organization, working memory, cognitive flexibility, and inhibition (e.g., Raboutet et al. 2010; Sergeant et al. 2002; Troyer et al. 1997; Unsworth et al. 2011). For that reason, they have proven valuable in identifying cognitive deficits associated with neurological and psychiatric disorders, monitoring disease progression, and evaluating the effectiveness of relevant interventions (e.g., da Silva et al. 2004). Alterations in verbal fluency performance have been observed in conditions such as aphasia, schizophrenia, epilepsy, Alzheimer’s, or Parkinson’s disease in adults (Bose et al. 2022; Carpenter et al. 2021; Chrobak et al. 2022; Robert et al. 1998; Tallberg et al. 2010; Yang et al. 2022), and attention deficit hyperactivity disorder (ADHD), Down syndrome, autism spectrum disorder (ASD), developmental language disorder (DLD), or dyslexia in children (e.g., Begeer et al. 2013; Koren et al. 2005; Mengisidou et al. 2020; Raboutet et al. 2010; Ralli et al. 2021; Troyer et al. 1997).

At the same time, verbal fluency tasks permit the study of age-related cognitive changes in typical populations (Álvarez Medina et al. 2023; Koren et al. 2005; Raboutet et al. 2010; Troyer 2000). In adults, a decline in verbal fluency performance is associated with aging (e.g., Rodríguez-Aranda and Martinussen 2006), whereas in children, a linear increase in the number of words retrieved is associated with language, memory, and executive function development (e.g., Villalobos et al. 2022).

### 1.1. Clustering, Switching, and Underlying Cognitive Processes

Scientific research on verbal fluency has flourished over the last decades, contributing valuable insights into the cognitive processes and neural mechanisms underlying performance in these tasks. Verbal fluency performance, predominantly measured by the total number of correct words produced during the time constraints of the task, has been associated with individual differences in linguistic knowledge, working memory, various dimensions of executive functioning, or even novel problem-solving and fluid intelligence (e.g., Abwender et al. 2001; Amunts et al. 2021; Fossati et al. 2003; Resch et al. 2014; Whiteside et al. 2016).

In an attempt to explain the processes underlying the link between the number of retrieved words and higher-order cognitive functioning, Troyer et al. (1997, 1998) proposed that two strategic processes, clustering and switching, are crucial for verbal fluency and affect performance in the task. The process of *clustering* involves accessing a subcategory of words stored in long-term memory and retrieving as many exemplars as possible from that subcategory (Robert et al. 1998; Troyer 2000; Troyer et al. 1997). Clustering can be observed when an individual’s output contains consecutive words belonging to the same subcategory (Troyer et al. 1997) and is typically quantified by calculating the mean cluster size, i.e., the mean number of words that the clusters produced during the task contain (Hurks et al. 2010; Kavé et al. 2008; Sauzéon et al. 2004; Tallberg et al. 2010). Cluster *switching*, on the other hand, involves shifting to a new subcategory when words from the previous one are exhausted or their retrieval becomes particularly difficult or slow (Hurks et al. 2010; Troyer 2000; Troyer et al. 1997; Wixted and Rohrer 1994). There have been several proposals on the measurement of switching, but the majority of studies quantify the ability to switch between subcategories by reporting on the number of switches and the number of clusters produced. More specifically, any change of subcategory (between two consecutive clusters, a cluster and a single word, or between two single words of a different subcategory) is counted as a switch (Troyer et al. 1997). An alternative measure of switching that does not give credit to single unclustered words and therefore is considered more robust (Hurks et al. 2010; Kavé et al. 2008; Koren et al. 2005) consists of counting the number of clusters generated and therefore giving credit only to the strategically performed switches/cluster initiations and not to randomly produced unclustered words.

One should note that clustering and switching strategies are not only complementary but also dissociable and, in a sense, ‘antagonistic’. Participants who manage to retrieve many words from just a few subcategories (e.g., participants with larger lexicons) and, therefore, make larger clusters would be expected to score lower in the number of switches or the number of clusters they produce within the time constraints of the task; and, vice versa, smaller clusters would call for the need to make more frequent switches to produce the same number of words. Clustering is proposed to be mainly affected by lexico-semantic knowledge and verbal memory and is found to be more associated with temporal lobe functioning (N’Kaoua et al. 2001; Testa et al. 1998; Troyer et al. 1998). Switching, on the other hand, is proposed to reflect a more controlled process linked with higher-order executive processes (e.g., initiation, working memory, set-shifting, cognitive flexibility) and is found to be mainly associated with frontal lobe functioning (Randolph et al. 1993; Testa et al. 1998; Troyer et al. 1998; Tröster et al. 1998; Woods et al. 2004). Although both clustering and switching are found to explain word productivity in verbal fluency tasks, performance in PVF has been shown to be mainly affected by switching, while in SVF, performance has been related to both clustering and switching strategies (Troyer et al. 1997).

### 1.2. Development of Clustering and Switching in Typically Developing Children

Despite the important theoretical interest in these more qualitative indices of verbal fluency performance, the vast majority of studies on clustering and switching have been conducted with adult samples, either healthy (see Oberg and Ramírez 2006) or clinical (see Metternich et al. 2014). Research on the development of these strategic processes in typically developing children and adolescents has been relatively neglected. In their recent systematic review, Arán Filippetti and colleagues (2022) retrieved 33 studies reporting on clustering and switching development in children, among which only 15 were on typically developing children and adolescents. On average, each of these studies covered a mean age span of 6.5 years, whereas there was considerable variability in the exact ages of the samples included in each study as well as in the languages they spoke (English, French, Spanish, Malayalam, Hebrew, Dutch, Portuguese, and Swedish), factors that are both found to affect the results significantly, as explained later.

Based on these studies, verbal fluency performance in typically developing children and adolescents seems to improve with age and to be more strongly associated with switching than clustering (e.g., Arán Filippetti and Allegri 2011; Sánchez-López et al. 2021; Tallberg et al. 2010). Most studies failed to record significant age-related changes for the mean cluster size, suggesting that clustering—primarily related to lexico-semantic knowledge, as mentioned earlier—is an easier, perhaps automatic, or earlier emerging strategy than switching. Given the minimal number of studies in very young children (i.e., only two studies including preschoolers in their samples), no safe conclusions can be drawn regarding its emergence.

Among the two switching factors, both the number of switches and the number of clusters were found to be strongly related to word productivity in SVF, whereas in PVF, the contribution of the number of switches appeared to be more pronounced (e.g., Becker et al. 2019; Kavé et al. 2008; Nieto et al. 2008; Sánchez-López et al. 2021). Switching increases with age, presumably due to the gradual maturation of higher-order/frontal lobe executive processes (Anderson 2002; Anderson et al. 2001; Koren et al. 2005). Indeed, two studies measuring simultaneously other cognitive and executive processes in children (Arán Filippetti and Allegri 2011, 8–11 years old; Becker et al. 2019, 6–12 years old) confirmed that the association of inhibition, working memory, and cognitive flexibility with the number of clusters and switches is more robust than their association with the mean cluster size. However, further studies covering a more extensive age range are needed to confirm these first results.

### 1.3. The Effect of Contextual Factors

Apart from age, the impact of contextual factors on children’s verbal fluency performance has received very limited attention. With the exception of data on the effect of children’s and adolescents’ sex/gender, we know very little about how other factors may affect their performance in fluency tasks and their clustering and switching strategies.

Regarding the effect of sex/gender on clustering and switching performance, the results are conflicting. Weiss et al. (2006) have shown that (adult) women tend to switch more often in the PVF, and men tend to produce larger clusters. Limited data on children confirms the tendency of girls to produce more clusters, this time in SVF tasks (Koren et al. 2005). Other studies, however, failed to find significant differences in children (e.g., Hurks et al. 2010; John et al. 2016). As Arán Filippetti et al. (2022) summarize in their systematic review, most studies found no differences between girls and boys, regardless of country of origin or language. Only two studies reported a sex effect on semantic cluster size, phonemic switches, and the number of switches when interacting with parental education (Arán Filippetti et al. 2022).

Also, some studies reported no sex/gender effect on the total number of words (e.g., Hurks et al. 2006; Kavé 2005; Van der Elst et al. 2011). Other studies reported a small female advantage in SVF (Olabarrieta-Landa et al. 2017; Koren et al. 2005; Rivera et al. 2021) or even others found that boys outperform girls (Ardila et al. 2005; Prigatano et al. 2008). In a recent meta-analysis, Hirnstein et al. (2023) analyzed data from 168 studies on children, adolescents, and adults. They concluded that girls/women outperform men/boys in PVF with a small effect size but not in SVF, where no sex/gender-related differences were found. They reached similar conclusions by presenting data separately for children (from preschool to 12 years) and adolescents (13–18 years).

The effect of education has been extensively studied in adult samples. Higher levels of education have been reported to be both related (e.g., Fichman et al. 2009; Kosmidis et al. 2004; Rosselli et al. 2009; Troyer 2000) and unrelated (e.g., Bolla et al. 1990; Shao et al. 2014) to higher scores in the verbal fluency task. These controversial findings could possibly be attributed to the different ages of the participants as well as to different cultural backgrounds. Studying two groups of different educational levels (literate and illiterate) but with the same socio-cultural background and similar age, da Silva and colleagues (2004) confirmed the strong effect of education on verbal fluency. The effects of literacy were also significant on the clustering and switching aspects of SVF, with literate people producing a larger number of switches and smaller clusters.

In children, few studies investigated the effect of parents’ educational level and found that a higher level of parental education is associated with children’s better performance in verbal fluency (Aksamovic et al. 2019; Ardila et al. 2005; Hurks et al. 2006; Van der Elst et al. 2011). As for the effect of parental education on clustering and switching strategies, a positive effect of parental education on mean cluster size (a lexico-semantic measure) but not on the number of clusters or the number of switches (executive functioning measures) is reported (Hurks et al. 2010). In their recent study on 4373 children from 9 countries, Olabarrieta-Landa et al. (2017) showed that other factors mediate the effect of parental education; the cultural context and the exact nature of verbal fluency tasks (SVF or PVF tasks) seem to play an important role in the impact of parental educational level.

Differences in verbal fluency performance have also been attributed to the properties of the language in which testing takes place. Although there are no comparative data on children, we know from adult studies that differences in word length from language to language, differences in category sizes (e.g., the category of vegetables includes all plants in Spanish but not in English) (Rosselli et al. 2002), and the phonemic system of a language (e.g., in Spanish, many words starting with the sound/a/are written as ha-) (Villalobos et al. 2022) influence performance in verbal fluency tasks. In their recent systematic review, Villalobos et al. (2022), by comparing the highest scores reported in 15 different languages further support cross-linguistic differences in fluency tasks.

### 1.4. Aims of This Study

All previously presented evidence suggests that, apart from the total number of words produced during a verbal fluency task, a qualitative analysis of the retrieval strategies implemented during a verbal fluency test can provide very useful insights into the cognitive processes underlying the rapid search and retrieval of words. Clustering and switching, thus, could be used to measure these underlying processes while assessing children’s cognitive development and account for verbal fluency development.

However, few studies have reported on the development of those cognitive strategies and their relationship with word productivity in typically developing children. Even fewer studies have covered a broad developmental period from preschool to adolescence or have reported on the effect of contextual factors (e.g., sex/gender or parental education) in this relationship. Finally, as far as we know, relevant data on clustering and switching from Greek-speaking participants are only available for adult samples (e.g., Kosmidis et al. 2004) but not for typically developing children or adolescents.

Based on data from an ‘animals’ SVF task administered to a wide sample of participants covering the age range from 4;00 to 16;11 years, the present study aims to investigate: (a) the development of the number of correctly retrieved words in Greek-speaking children and adolescents; (b) the development of the clustering and switching strategies employed; (c) the effect of children’s sex on word productivity, clustering, and switching; (d) the effect of parental education on word productivity, clustering, and switching; and (e) the combined effect of all these factors on children’s performance in the task. A complementary methodological aim of this study is to explore the development and effect of a new clustering measure, that of the maximum cluster size (see Section 2.4).

## 2. Materials and Methods

This study used data (word lists and audio recordings) from a SVF task administered to the participants of a larger research project that focused on the mechanisms underlying cognitive development in children and adolescents. For the specific needs of the present study, these data were further analyzed and coded, including measurements of clustering and switching.

This study has a cross-sectional design with four measures of clustering and switching as predictor variables and the number of correct words retrieved during the SVF task as a criterion variable. Based on previous literature, four additional predictors—children’s age and sex, and maternal and paternal education—were also measured and used to control for the effect of contextual factors.

### 2.1. Participants

The sample consisted of 472 children (51.1% girls) ranging from 4;0 to 16;11 years of age. Table 1 presents the sample distribution according to age and sex, and Table 2 presents its distribution according to parental educational level. All children were monolingual Greek-speaking, and Greek was the only language spoken in their homes. Children with a diagnosed sensory, physical, cognitive, or language impairment were not included in the sample.

### 2.2. Task and Materials

All children were administered an ‘animals’ SVF task (e.g., Troyer et al. 1997). The exact instructions given to participant children were, “I want you to tell me in one minute as many animals as you can think of. Go as fast as you can. Ready? Go!”

In addition, their parents filled out a background questionnaire, providing demographic data on the children and their families and information on the languages spoken at home or potential diagnoses of impairments or difficulties.

### 2.3. Procedure

The research was conducted following an ethics clearance by the Research Ethics Committee of the Department of Primary Education of the Democritus University of Thrace (Ref. ΔΠΘ/ΠΤΔΕ/62180/1866/2020) and permission from the Ministry of Education to access public schools (Φ15/110217/ΕΚ/126648/Δ1). Participants’ data were treated anonymously throughout the research. The consent of the Director of each school was granted, followed by the informed consent of the children’s caregivers and the class teachers.

Children were tested individually in a stimulus-free room at their school. Testing was conducted by five well-trained research assistants (doctoral and graduate students). After a time of habituation with the research assistants, instructions were given to the children, and the testing time began. The 60 s time limit was kept with the help of a stopwatch. While children were tested, the research assistants took note of the words they produced, including repeated words (*perseverations*) or non-animal words (*intrusions*). Each session was also audio-recorded. The research assistants’ notes, as well as the duration of the testing time, were later checked for accuracy by two independent scorers (k = .93).

### 2.4. Verbal Fluency, Clustering, and Switching Scoring

Analyses were aided by the Python *Semantic Network and Fluency Utility* library (SNAFU; Zemla et al. 2020), a tool for the automated analysis of fluency data developed to guarantee transparency and reproducibility of verbal fluency results. For that purpose, a *data file* with each child’s words (including perseverations and intrusions) was produced according to the specifications of SNAFU.

For the purpose of the analysis, a semantic *scheme file* for Greek animal words had to be produced. The semantic scheme comprised 380 different animals organized into 32 subcategories based on zoological categories (e.g., birds, reptiles), habitat (e.g., water, farm), continent (e.g., Africa, Australia), appearance (e.g., animals with horns), and human use (e.g., beasts of burden, pets). Mythical or extinct animals (e.g., dinosaurs, unicorns) were also included in a separate subcategory. Each animal could belong to more than one subcategory, e.g., dog—pets; dog—canines.

In addition, a *spelling file* was produced, including all word forms that should be identified as the same word (e.g., the same word in a different grammatical case, number or gender, diminutives, exact synonyms, or common spelling errors). This file contained 714 different pairs of equivalent forms.

After running preliminary analyses, a detailed manual inspection of the results for potential errors was carried out. After making minor corrections to the scheme and the spelling files and re-running the analyses, post hoc inspections of the data confirmed that no animal words were characterized as intrusions, that no two versions of the same word were identified as two different words, and that clusters were correctly demarcated. The scheme and spelling files used for the analyses, together with instructions for preparing Greek font data to be readily analyzed with SNAFU, are available at Karousou and Thomaidou (2023).

The score of the SVF task (number of correct words, NCW) was automatically calculated by SNAFU by subtracting the occasional perseverations (repeated words) and intrusions (non-animal words) from the total number of words produced by each child. As for the demarcation of the clusters, in line with Troyer et al. (1997), clusters were defined as successively generated words belonging to the same subcategory. Each cluster was taken to begin at the termination of a previous cluster or after an unclustered word and to end when the next word did not belong to the same subcategory (a *static* cluster in SNAFU).

In order to gain more control over the analyses of the clustering and switching variables, we used the output of SNAFU reporting the clusters each child generated (quantified in SNAFU as 1 for unclustered single words, 2 for two-word clusters, etc.) to calculate the following clustering and switching variables.

Two variables were calculated as measures of switching:

- **Number of switches**: The number of switches was calculated by counting the number of transitions between clusters, including single words.
- **Number of clusters**: The number of clusters of at least two consecutive words belonging to the same subcategory was recorded.

Two variables were calculated as measures of *clustering*:

- **Mean cluster size**: The average number of words belonging to a cluster. In line with Troyer et al. (1997), perseverations and intrusions were included.
- **Maximum cluster size**: The number of words produced within the largest cluster each child produced.

Note that, as far as we know, no study has previously reported on the *maximum cluster size*. We take this variable to reflect (and quantify the maximum extent of) the participants’ ability to spontaneously deploy and maintain a clustering strategy without being affected by whether they fail, for any reason (e.g., lexico-semantic limitations), to implement it consistently with all subcategories.

## 3. Results

### 3.1. Number of Correct Words and the Effect of Age, Sex, and Level of Parental Education

**Figure 1:**
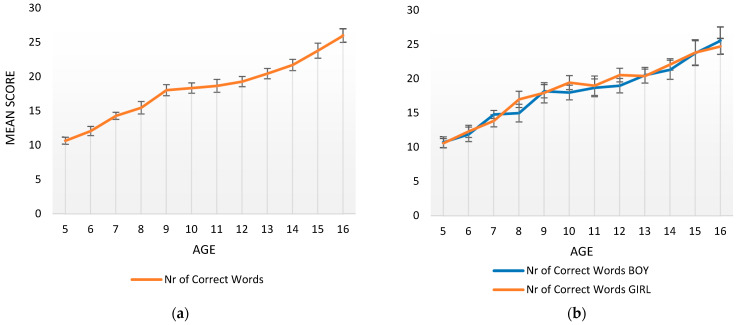
(**a**). Mean and Standard Error in the number of correct words retrieved per year of age. (**b**). Mean and Standard Error in the number of correct words retrieved per year of age and sex.

The first group of analyses presents the development of the SVF score and examines the effect of age, sex, and parental education on children’s word productivity (NCW: number of correct words). Figure 1a presents the NCW produced by the participants per year of age, and Figure 1b presents the NCW produced by the participants per year of age and sex.

As illustrated in Figure 1a, a linear increase in the NCW is observed, starting from a mean of 8.22 words at 4;00 years to reach a mean of 25.32 words by 16;11 years. An analysis of variance confirmed, as expected, a large effect of age on the score of the SVF task [*F*(11, 1) = 39.76, *p* = .000, η^2^ = .510].

Children’s sex did not seem to affect the results (see Figure 1b). A *t*-test analysis between boys (mean NCW = 17.50) and girls (mean NCW = 18.06) indicated no significant differences in children’s NCW [*t*(470) = .898, *p* = .370, *d* = .083].

As for the level of parental education, both maternal and paternal education were found to affect children’s NCW significantly, albeit with a small effect size [*F*_mother_ (2, 442) = 3.84, *p* = .022, η^2^ = .017 and *F*_father_ (2, 441) = 3.79, *p* = .023, η^2^ = .017]. Post hoc analyses showed a significant difference between the scores of children whose parents completed only the 9-year compulsory education and those with a university degree.

### 3.2. Clustering and Switching Strategies and the Effect of Age, Sex, and Parental Education

**Figure 2:**
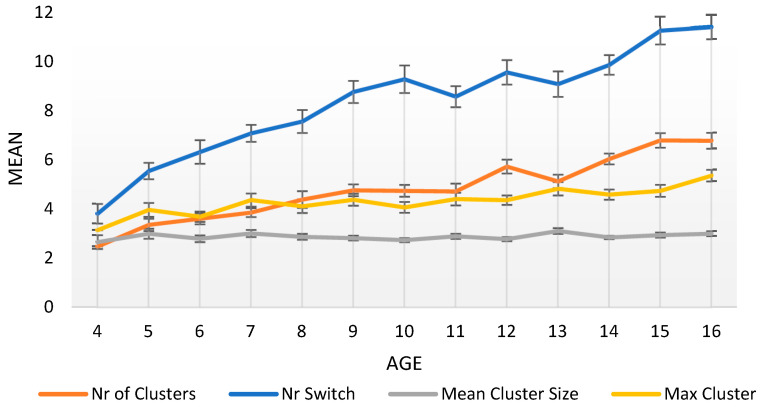
Mean and Standard Error in clustering and switching measures per age.

In this second set of analyses, we examine the development and the effect of age, sex, and level of parental education on the strategic processes of clustering and switching. Figure 2 represents the development of the clustering and switching measures across the participants’ age span, and Table 3 presents the results of a series of analyses of variance with age, sex, and parental education as independent variables and each strategy examined as a dependent variable.

Among the four clustering and switching measures studied, only one did not present a significant increase across the years. More specifically, the two switching factors, measuring the number of clusters and the number of switches between consecutive words of different subcategories, presented a significant increase, with age affecting these two factors with a major effect size. As for the effect of age on clustering, it did not significantly affect the mean cluster size, which remained practically stable across time. However, age had a significant effect, albeit moderate, on the new cluster size factor we measured, the maximum cluster size.

Finally, concerning the effect of children’s sex, although girls tended to produce slightly more switches than boys, and boys to produce slightly larger clusters than girls, after correcting the significance level with a Bonferroni procedure due to multiple comparisons, children’s sex did not appear to affect their clustering or switching scores. A lack of effect was also found for parental educational level.

### 3.3. The Relationship between Clustering and Switching Strategies and the Number of Correct Words

In this set of analyses, we examine the associations among the four strategic factors and the number of different words children produced correctly (NCW). Given the large effect of age on both the NCW and several of the cognitive strategic factors, as well as the effect of parental education on the NCW, a partial correlation controlling for the participants’ age and parental education was also examined. Table 4 presents these correlations.

The NCW was significantly correlated with all clustering and switching factors, even when age, sex, and parental education effects were controlled. The lowest amount of shared variance in the NCW was evidenced by the mean cluster size (*r*^2^_partial_ = .03), followed by the maximum cluster size (*r*^2^_partial_ = .17), the number of switches (*r*^2^ = .44), and the number of clusters (*r*^2^_partial_ = .51).

As for the partial correlations among the four strategic retrieval factors, they were all significantly correlated, although, as expected, the correlation of the number of clusters and the number of switches with the mean cluster size was negative (and low; *r*^2^_partial_ = .03 and *r*^2^_partial_ = .08, respectively). The new measure of maximum cluster size also had a negative -albeit very low- correlation with the number of switches (*r*^2^_partial_ = .01); however, its correlation with the number of clusters was positive -again low (*r*^2^_partial_ =.01). A moderate correlation was also found between the two clustering factors (mean and maximum cluster size) (*r*^2^_partial_ = .34) and also between the two switching factors (number of clusters and number of switches) (*r*^2^_partial_ = .21).

### 3.4. The Effect of Clustering and Switching, Age, Sex, Maternal, and Paternal Education on Word Productivity

Initially, a series of simple regressions (Table 5) were carried out, with NCW as a dependent variable, to determine the factors that would be entered in the multiple regression models. The individual effect of all factors, except for sex, on the NCW was shown to be significant.

Next, a series of hierarchical multiple regression analyses were conducted to examine the effect of the clustering and switching factors on the NCW in interaction with age and the level of parental education. Sex was excluded from analyses as it was found to have no significant effect.

Aiming to measure first the individual effect of each strategic factor on the NCW in interaction with children’s age and parental education, four hierarchical multiple regressions were initially conducted, in which each clustering and switching factor was entered on its own in a first block, age was entered in a second block, and maternal and paternal education were entered in a third block (see Table 5, *Individual effects*). The Variance Inflation Factor (VIF) ranged between 1.000 and 1.526, indicating small correlations among predictors in all regression models. A global hierarchical regression was conducted next, aiming to measure the combined effect of all factors on NCW (see Table 5, *Combined effects*). In that case, the Variance Inflation Factor (VIF) ranged between 1.010 and 2.326. Further collinearity diagnostics (Condition Index and Variance Proportions) confirmed that multicollinearity did not exceed acceptable levels (e.g., Kutner et al. 2005).

Table 6 summarizes the regression results. Each model’s regression statistics are presented separately, together with the change statistics (Δ*R*^2^, Δ*F*, and Sig. Δ*F*) after entering each block of predictors. All models were found to be significant, and all four factors appeared to affect children’s word productivity significantly. However, the effects of the two switching factors exceeded those of the clustering factors.

More specifically, the individual factor with the largest effect was the **number of clusters** (Model 1), which predicted 68.6% of the variance in the NCW score. The inclusion of children’s age enhanced the predictive capacity of the model by an additional 6.3%, and parental education (mainly maternal education) contributed an additional 05%. Overall, 75.4% of the SVF score could be predicted by the number of clusters in interaction with children’s age and parental education.

The individual effect of the **number of switches** (Model 2) was also very significant, though somewhat lower, as it was found to predict 61.2% of the variance in NCW. Age, in this case, increased the model’s predictive capacity by 9.8%, and parental education contributed an additional .08%, again with maternal education mainly contributing to this effect. The number of switches interacting with children’s age and parental education predicted 71.8% of the SVF score.

Among the two clustering factors, the one that presented the largest individual effect was the **maximum cluster size** (Model 3), which predicted 22.9% of the variance in NCW. Children’s age yielded an additional contribution of 34% to the model’s predictive capacity, and parental education—mainly due to the significant effect of maternal education—added an additional 1.2%. The maximum cluster size interacting with children’s age and parental education was found to predict 58.1% of the SVF score.

Finally, the smallest individual effect on NCW was evidenced in the **mean cluster size** (Model 4), which only predicted 2.1% of the variance in word productivity. Age added 47% over and above the effect of mean cluster size, and parental education contributed an additional 1.9%. The mean cluster size, interacting with children’s age and parental education, predicted 51% of the variance in the NCW.

As for the combined effect of all strategic factors on the NCW (Model 5), the four clustering and switching factors together predicted 90.3% of children’s word productivity. Controlling for children’s age added a significant 1.1% to the model’s predictive capacity, while maternal and paternal education enhanced the model by a non-significant .01%. All the factors together appeared to explain 91.4% of the variance in the SVF score.

## 4. Discussion

This study aimed to explore the development of the clustering and switching strategies employed by Greek-speaking children and adolescents while trying to retrieve as many animal words as they could in a SVF task. At the same time, the association of these cognitive strategies with the number of words children managed to produce was explored, as well as the effect of children’s sex and their parents’ education level.

We opted for the administration of a SVF task using the category ‘animals’ for several reasons. Among the various categories that can be used in a verbal fluency task, the ‘animals’ category is found to present minor differences among people living in different countries, speaking different languages, being exposed to different educational systems, or belonging to different generations (Ardila 2020). Moreover, as Ardila et al. (2006) conclude, the ‘animals’ SVF task fulfills four fundamental criteria that are important in a robust neuropsychological test: it has the largest normative database (different ages, education levels, and cultures), we know which brain areas are involved in its performance, how the test performance is affected in cases of pathology, as well as how it correlates with other cognitive neuropsychological measures. In addition, animal words are among the earliest emerging words in children’s vocabularies, irrespective of the language they speak (Frank et al. 2017), so using the ‘animals’ category, unlike other categories, is meaningful for testing young children. Finally, it is the category used by most studies exploring clustering and switching in children, either as a unique measure of verbal fluency (*n* = 14) or in conjunction with other measures (*n* = 12) (Arán Filippetti et al. 2022).

The present study reported, as far as we know for the first time, results on the development of SVF and the cognitive strategies of clustering and switching in typically developing Greek-speaking children and adolescents. Despite the frequent use of verbal fluency tasks in Greek studies, most of them include exclusively adult samples—typical or atypical (Andreou and Trott 2013; Bozikas et al. 2005; Karaivazoglou et al. 2007; Konstantopoulos et al. 2014; Kosmidis et al. 2005; Peristeri and Tsapkini 2011; Stavroussi et al. 2016). Normative data for SVF and PVF tasks, including clustering and switching measures, also concern Greek adults (Kosmidis et al. 2004). Finally, as far as we know, four studies have used verbal fluency tasks for the assessment of young Greek-speaking participants; however, they mainly focused on atypical development or did not analyze the clustering and switching strategies employed by the children (Konstantopoulos et al. 2014; Mengisidou 2019; Ralli et al. 2021; Zarokanellou et al. 2022).

To our knowledge, this is also the first study on the development of SVF, clustering, and switching that covered a 13-year developmental period, from preschool (4;00 years) to adolescence (16;11 years). The results we presented illustrated a continuous development of word productivity, with age exerting a significant effect on the NCW. Contrarily to some studies suggesting that verbal fluency reaches adult levels around the age of 10 (e.g., Anderson et al. 2001; Regard et al. 1982) or some studies pointing to a plateau performance around 12–13 years for SVF (Martins et al. 2007; Sauzéon et al. 2004), our findings support the results reported for Hebrew (Kavé 2006), Finnish (Klenberg et al. 2001), or Spanish-speaking children (Matute et al. 2004), showing that SVF continues developing during adolescence. For instance, Kavé (2006) found that their adolescent sample did not reach adult performance even at 16–17 years. In our case, we cannot know whether the 25.32 words produced on average by our 16;00–16;11 years group correspond to adult performance or not, since no comparative data are available for an ‘animals’ SVF task in Greek typical adult samples (norming and other data report only the sum of three semantic categories, mainly animals, fruit, and objects, e.g., Kosmidis et al. 2004). A future study could extend the age span of our study to include a Greek-speaking adult ‘control’ sample aiming to address this gap.

In any case, since SVF performance has been associated with and explained by participants’ ability to employ clustering and switching strategies for word retrieval (Troyer 2000; Troyer et al. 1997, 1998)—a finding also supported by our data, as will be discussed later—results on the protracted development of word productivity could be better explained by looking into the development of the clustering and switching strategies measured in the present study.

Confirming previous relevant research findings on clustering and switching in children (e.g., Becker et al. 2019; Sánchez-López et al. 2021), the NCW produced during the task was found to be associated both with the two switching factors and the two clustering factors. However, also in line with the vast majority of relevant studies (for a systematic review, see Arán Filippetti et al. 2022), the largest amount of variance in word productivity was found to be attributed to switching, the ability to shift into different semantic subcategories in order to search more effectively for additional items within the semantic network. In line with several other studies (e.g., Arán Filippetti and Allegri 2011; Hurks et al. 2010), considering that both the *number of clusters* and the *number of switches* may represent indicators of strategic retrieval processes and cognitive flexibility, we used both measures to quantify switching. The correlation between the two switching factors was found to be moderate. Both factors were associated with the NCW with a very large effect size, with the number of clusters explaining word productivity slightly better (69.8%) than the number of switches (62.2%). According to several authors (e.g., Hurks et al. 2010; Kavé et al. 2008; Koren et al. 2005), the number of clusters, by excluding non-strategic switches that are due to unclustered words and, thus, being more independent of the total NCW, is considered to be a more robust measure of switching than the number of switches. Apart from being theoretically more robust, we confirmed that it is also statistically more robust in explaining the SVF score.

Regarding their development, confirming previous studies that reported age-related increases in switching (e.g., Kavé et al. 2008; Koren et al. 2005; Tallberg et al. 2010), both switching factors presented a significant increase throughout the developmental period studied. Since this is the first study to report on such an extensive developmental period, this finding is particularly interesting. Switching has been associated with higher-order frontal lobe-mediated executive processes (e.g., Randolph et al. 1993; Testa et al. 1998; Troyer et al. 1998; Tröster et al. 1998; Woods et al. 2004) that include initiation, set-shifting, monitoring, and inhibition, and its development has been linked to the gradual development of several executive functions (Anderson 2002; Anderson et al. 2001; Koren et al. 2005), including cognitive flexibility and set-shifting (Hurks et al. 2010; Kavé et al. 2008; Troyer et al. 1997) but also inhibition and working memory (Arán Filippetti and Allegri 2011; Becker et al. 2019). It is known that basic skills needed for executive functioning (EF) begin to emerge early in infancy, and more specific skills develop into early childhood. Although each component of EF develops at its own rate across childhood and adolescence, we know that more complex EF skills of working memory, inhibition, and shifting continue developing through adolescence until at least early adulthood (e.g., Boelema et al. 2014; Gur et al. 2012; Huizinga et al. 2006; León-Carrión et al. 2004; Zelazo et al. 2003; for recent reviews, see Best and Miller 2010; Ferguson et al. 2021; Theodoraki et al. 2020). A future study could attempt to disentangle the relative contribution of each EF skill in the different periods of switching development and offer an in-depth account of the underlying factors explaining its protracted development.

Concerning the results for the two clustering factors studied, the *mean cluster size* remained practically stable, presenting no significant changes during the entire age span of the present study. Our results, thus, confirmed those of the vast majority of earlier studies (see Arán Filippetti et al. 2022), which suggested that clustering—a strategy primarily related to lexico-semantic knowledge—is an easier, perhaps automatic or earlier emerging strategy than switching. Overall, the mean cluster size was found to be the factor with the poorer association with the NCW children managed to produce, significantly predicting only 2.4% of the variance in word productivity. The mean cluster size was also found to have negative correlations with the two switching factors, confirming the ‘antagonistic’ (inverse proportional) nature of this relationship; as expected, children with overall smaller clusters were found to make more switches between words of different subcategories.

As for the earlier emergence hypothesis, only two studies measured the mean cluster size in children younger than 5 years apart from the present study. Resch et al. (2014) studied Dutch-speaking children aged 4–5 and 5–6 years and found a significant difference in the mean cluster size between the two groups (however, no descriptive results are presented to compare with our results). Chami et al. (2017), based on a sample of 4- to 10-year-old Australian-English-speaking children, found significant differences in the mean cluster size only between the 4- and 5-year-old groups. Although the mean cluster sizes found in Chami et al. are comparable to ours, we failed to detect significant differences between 4- and 5-year-old children (mainly because our 4-year-old sample scored slightly higher). The early emergence or automaticity of the clustering strategy during verbal fluency tasks should be systematically addressed in future studies, including perhaps even younger children in their samples.

Adopting an alternative approach to measuring children’s clustering ability, however, we introduced a second measure of clustering, the *maximum cluster size*. We assumed it could be a qualitatively different measure since, unlike the mean cluster size, which considers the average size of all clustering attempts participants initiate, the maximum cluster size reflects each participant’s competence in clustering, regardless of whether this competence is materialized consistently with all subcategories accessed during the task. The production of some small clusters is not necessarily linked to a lack of the corresponding strategic/cognitive tools to generate multi-word clusters; lexico-semantic limitations in certain subcategories (e.g., young children may know many farm animals but not as many names of birds), subcategories that inherently include few animals, or animals with a lower frequency of use, could cause the production of small clusters and affect the mean cluster size. We, therefore, took the maximum cluster size as a way to quantify the participants’ capacity to deploy and maintain a clustering strategy, not affected by incidental activation of subcategories in which children would not have the necessary ‘materials’ to implement a large cluster. In that sense, we assumed that the maximum cluster size could be found to be less affected by the participants’ lexico-semantic knowledge and, thus, reflect a more cognitive dimension of clustering than the mean cluster size.

The maximum cluster size was found to have a moderate correlation to the mean cluster size, providing evidence of the fact that it measures a related, albeit different/complementary dimension of clustering. Interestingly, the results on its development, unlike those of the mean cluster size, revealed a small but significant effect of age; children’s competence in clustering several words of the same subcategory gradually improved with age. In addition, the maximum cluster size was found to predict a significant proportion (23.6%) of the variance in the NCW children produced. Whether the maximum cluster size is less affected by children’s lexico-semantic knowledge and more linked to cognitive processes is an important issue that will have to be addressed in future studies simultaneously measuring children’s lexical and other cognitive skills.

Overall, the four clustering and switching factors taken together had a very large combined effect on children’s word productivity and explained 90.3% of the variance in the NCW. The overall effect of these factors, when combined with the effect of age and that of parental education, accounted for 91.4% of the variance in the score in SVF.

Concerning the effect of the contextual factors measured in this study, children’s sex was not found to affect word productivity. This finding is in line with the recent systematic review of Hirnstein et al. (2023), who, after analyzing the results of 168 studies, concluded that there are no sex/gender-related differences in SVF task performance, neither in adults nor in children or adolescents. Furthermore, our results did not find significant sex-related differences in clustering and switching either, further supporting relevant findings of other studies (e.g., Hurks et al. 2010; John et al. 2016). More specifically, the number of clusters and the maximum cluster size did not present any difference between boys and girls. As for the number of switches and the mean cluster size, although girls were found to have a tendency for a slightly higher number of switches and boys for a slightly higher mean cluster size, these differences had a low effect size and, after correcting the level of significance, were not deemed significant. However, these same tendencies were also detected in earlier studies with children and adults. Weiss et al. (2006) found that (adult) women tended to switch more and men tended to produce larger clusters; Koren et al. (2005) detected a tendency for girls to produce more clusters. More empirical studies will be needed to support these tendencies and try to offer a meaningful interpretation.

As for the two parental education factors, both maternal and paternal education were found to affect the NCW, although the amount of variance in word productivity they explained did not exceed 1.5% in either case. However, neither maternal nor paternal education appeared to have a significant effect on the clustering and switching strategies, while only maternal education in interaction with children’s age and the clustering and/or switching strategic factors seemed to predict the NCW with a very low effect size. Previous results on the effect of parental education have been contradictory. Some studies on children of different ages (Aksamovic et al. 2019; Ardila et al. 2005; Hurks et al. 2006, 2010; Van der Elst et al. 2011) have suggested that parental education mainly affects the NCW and the mean cluster size, which are linked to a large extent to children’s lexico-semantic knowledge. These findings are explained by studies on language development showing that children from higher-educated parents also tend to have larger vocabularies (e.g., Hart and Risley 1995; Hoff 2003). Contrarily, studies have generally failed to find a significant effect of parental education on the switching factors, which are thought to reflect higher-order cognitive processes (e.g., Hurks et al. 2010, but not Resch et al. 2014 with children aged 4–6). The fact that we did not find an effect of parental education on the mean cluster size is very difficult to interpret with the available data. Parental education is a factor whose effects are found to be mediated by several other factors of the family context in which children and adolescents develop (e.g., the socio-cultural context, the dynamics of family interactions and routines, parents’ beliefs on their role in enhancing language and cognitive development, the amount of time parents interact with their children, the quality of these interactions, the home learning environment, the availability of reading materials at home, the shared book reading quantity and quality) (e.g., Davis-Kean 2005; Hirsh-Pasek et al. 2015; Hoff 2003; Karousou and Economacou, forthcoming; Penderi and Papanastasatou 2022; Weigel et al. 2006). Further studies, focusing exclusively on the issue of the family context, should address the effect of the various contextual factors that may affect children’s verbal fluency development.

The results we presented have two main limitations that should be taken into account. The first is related to the fact that they stem from a cross-sectional study. Although we presented for the first time results from a very broad age range, permitting us to outline the development of a series of abilities as measured consistently—with the same methodological tools—from preschool to adolescence, cross-sectional data cannot provide detailed information about the individual trajectories in cognitive development and, therefore, they do not permit the extraction of in-depth accounts of the processes underlying developmental change or individual differences in development (e.g., Grammer et al. 2013; Magnusson and Stattin 2006). As far as we know, there have been no longitudinal studies reporting on the development of verbal fluency in children and/or adolescents, nor on their relationship with other cognitive/executive processes. We suggest that this is an important line of research to be pursued in the future.

Another issue is related to the educational level of the participating families. Less than 8% of the parents had completed only compulsory education, while approximately 40% of the fathers and 55% of the mothers had a university degree. The unequal representation of parents with very low educational levels might have led to an underestimation of the influence of these factors on the results. Future studies should aim at oversampling families of lower economic and educational strata to tackle this difficulty, but they could also take into account other factors of the family context that, as mentioned earlier, could be mediating the effect of parental education on verbal fluency development.

Despite these limitations, we believe that the results we presented offer a coherent picture of verbal fluency development that can serve as a solid empirical basis for further explorations and for formulating more specific theoretical hypotheses and expansions, addressing the specific knowledge gaps that still exist in the relevant literature.

## 5. Conclusions

Verbal fluency is considered an important process for language and cognitive function, as it reflects a person’s ability to rapidly access and retrieve lexical and semantic knowledge stored in long-term memory. As such, verbal fluency is an integral aspect of a person’s psychosocial functioning, affecting effective communication, efficient symbolic thinking, and emotional expression.

In spite of the simplicity of the semantic verbal fluency tasks, they are suggested to provide sensitive and accurate indexes of a person’s verbal fluency skills; thus, they are systematically used as diagnostic tools for a variety of clinical conditions, both in children and adults. The large body of empirical evidence summarized in this paper suggests that verbal fluency skills are not only affected by lexico-semantic knowledge or its organization in semantic networks but also, and most importantly, by core executive processes, such as working memory, inhibition, or set-shifting, which provide the cognitive tools for the successful search and retrieval of stored lexical entries. Apart from the total number of words a person manages to retrieve correctly within the time constraints of the task, the estimation of the strategic processes of clustering and switching has been proposed not only to explain performance in the task but also to be useful in measuring the underlying cognitive processes that enable fluent verbal production.

Despite the usefulness and extensive use of these measures for clinical purposes, however, it is striking how limited the available empirical basis is concerning their normative developmental trajectories and the factors that affect them. A little more than a dozen studies, varying greatly in the ages they cover and the languages on which they report, constitute the only evidence available on the subject.

The results we presented confirmed the importance of these qualitative measures in the assessment of verbal fluency in children. They presented, we believe, a coherent picture of the development of clustering and switching across a wide developmental period covering the preschool years, middle childhood, later childhood, and adolescence, and highlighted the role of these executive strategic processes in the successful retrieval and production of relevant lexical entries. However, we also highlighted several knowledge gaps that still exist in the understanding of the development of these processes in typically developing children that should be addressed in future studies for a deeper understanding of verbal fluency development. These gaps stem from the complete lack of longitudinal studies on the development of clustering and switching processes, the lack of neuro-imaging studies on the brain functions that support them during different developmental periods, the scarcity of data on how these strategic processes relate to the development of the executive functions they are suggested to reflect in different developmental phases, or the scarcity of data on factors of the context within which children grow up that affect their development. This knowledge is not only necessary for reaching an in-depth understanding of the processes that underlie developmental change in verbal fluency, for understanding the causes of individual differences in its development, or for detecting the developmental causes of verbal fluency deficits; it is also crucial for grasping the full nature of these processes and detecting the roots and causes of individual differences in verbal fluency performance, even in adults.

## Figures and Tables

**Table 1:** Distribution of children according to their age and sex.

Age (Years)	Boys		Girls		Total
	N	%	N	%	
4	15	48.39	16	51.61	31
5	19	52.78	17	47.22	36
6	19	52.78	17	47.22	36
7	20	46.51	23	53.49	43
8	16	48.48	17	51.52	33
9	19	43.18	25	56.82	44
10	20	50.00	20	50.00	40
11	14	48.28	15	51.72	29
12	20	52.63	18	47.37	38
13	14	46.67	16	53.33	30
14	20	47.62	22	52.38	42
15	17	47.22	19	52.78	36
16	20	52.63	18	47.37	38
**Total**	**233**	**48.94**	**243**	**51.06**	**476**

**Table 2:** Distribution of children according to parental educational level.

Educational Level	Father (%)	Mother (%)
University degree	38.8	53.0
Baccalaureate or technical school	53.6	40.0
Compulsory education	7.6	7.0

**Table 3:** Age, sex, and level of parental education (LPE) differences in clustering and switching measures.

Clustering and Switching Factors	AGE			SEX			LPE Mother			LPE Father		
	F	*p*	η^2^	*t* (Boys-Girls)	*p*	d	F	*p*	η^2^	F	*p*	η^2^
Nr of Clusters	17.60	.000 *	.309	−1.07	.283	−.102	2.25	.107	.010	3.76	.034	.017
Nr of Switches	16.18	.000 *	.292	−1.92	.049	−.180	2.16	.116	.010	2.81	.062	.013
Mean Cluster Size	1.06	.391	.026	2.33	.020	.222	0.74	.480	.003	0.10	.901	.000
Max Cluster Size	3.49	.000 *	.082	1.36	.175	.129	2.51	.082	.011	1.19	.304	.005

Note: After applying a Bonferroni correction, due to multiple comparisons, significant differences are marked with an asterisk (*).

**Table 4:** Correlations among clustering, switching measures, and the number of correct words.

Clustering and Switching Factors	NCW		Nr of Clusters		Nr of Switches		Mean Cluster Size	
	Pearson	Partial ^×^	Pearson	Partial ^×^	Pearson	Partial ^×^	Pearson	Partial ^×^
Nr of Clusters	.835 **	.715 **	--	--				
Nr of Switches	.788 **	.663 **	.646 **	.455 **	--	--		
Mean Cluster Size	.156 **	.169 **	−.103 *	−.174 *	−.196 **	−.278 **	--	--
Max Cluster size	.486 **	.411 **	.267 **	.114 *	−.094 *	−.092 *	.761 **	.580 **

** Correlation is significant at the .01 level. * Correlation is significant at the .05 level. ^×^ Partial correlations controlling for age, sex, paternal, and maternal education.

**Table 5:** Simple regressions: the effect of all factors on NCW.

	Factor	*R* ^2^	*F*	β	Sig
Clustering and Switching	Nr of Clusters	.698	1086.47	.835	.000
	Nr of Switches	.622	772.01	.788	.000
	Mean Cluster Size	.024	11.79	.156	.000
	Max Cluster Size	.236	145.15	.486	.000
Child	Age	.493	456.77	.702	.000
	Sex	.002	.806	.041	.370
Parents	Maternal Education	.013	5.87	.114	.016
	Paternal Education	.015	6.85	.124	.009

**Table 6:** Hierarchical regressions: the effect of clustering and switching, age, sex, and parental education on the number of correct words.

	Model *	*R* ^2^	*F*	Sig	Δ*R*^2^	Δ*F*	Sig. Δ*F*	BLOCK 1								BLOCK 2		BLOCK 3			
								Nr of Clusters		Nr of Switches		Max Cluster Size		Mean Cluster size		Age		Maternal Education		Paternal Education	
								β	Sig	β	Sig	β	Sig	β	Sig	β	Sig	β	Sig	β	Sig
**Individual effects**	1a	.686	958.62	.000	.686	958.62	.000	.828	.000	-	-	-	-	-	-	-	-	-	-	-	-
	1b	.749	653.49	.000	.063	109.94	.000	.646	.000	-	-	-	-	-	-	.310	.000	-	-	-	-
	1c	.754	333.25	.000	.005	4.01	.019	.635	.000	-	-	-	-	-	-	.318	.000	.063	.019	.010	.715
	2a	.612	691.85	.000	.612	691.85	.000	-	-	.783	.000	-	-	-	-	-	-	-	-	-	-
	2b	.710	534.72	.000	.098	146.99	.000	-	-	.578	.000	-	-	-	-	.373	.000	-	-	-	-
	2c	.718	276.56	.000	.008	6.05	.003	-	-	.567	.000	-	-	-	-	.381	.000	.084	.004	.011	.705
	3a	.229	130.25	.000	.229	130.25	.000	-	-	-	-	.479	.000	-	-	-	-	-	-	-	-
	3b	.569	288.16	.000	.340	344.06	.000	-	-	-	-	.317	.000	-	-	.605	.000	-	-	-	-
	3c	.581	150.77	.000	.012	6.34	.002	-	-	-	-	.305	.000	-	-	.608	.000	.077	.029	.053	.127
	4a	.021	9.49	.002	.021	9.49	.002	-	-	-	-	-	-	.146	.000	-	-	-	-	-	-
	4b	.491	211.16	.000	.470	404.11	.000	-	-	-	-	-	-	.125	.000	.686	.000	-	-	-	-
	4c	.510	113.38	.000	.019	8.42	.000	-	-	-	-	-	-	.120	.000	.687	.000	.107	.005	.051	.176
**Combined effect**	5a	.903	1006.93	.000	.903	1006.93	.000	.486	.000	.492	.000	.191	.000	.163	.000						
	5b	.913	914.37	.000	.011	53.94	.000	.436	.000	.449	.000	.177	.000	.153	.000	.136	.000				
	5c	.914	655.44	.000	.001	1.62	.200	.434	.000	.447	.000	.173	.000	.155	.000	.140	.000	.027	.088	−.004	.804

* Note. Dependent Variable: Number of Correct Words. **Model 1** Predictors in blocks: [Number of Clusters], [Age (in years)], [Maternal Education, Paternal Education]; **Model 2** Predictors in blocks: [Number of Switches], [Age (in years)], [Maternal Education, Paternal Education]; **Model 3** Predictors in blocks: [Max Cluster Size], [Age (in years)], [Maternal Education, Paternal Education]; **Model 4** Predictors in blocks: [Mean Cluster Size], [Age (in years)], [Maternal Education, Paternal Education]; **Model 5** Predictors in blocks: [Number of Clusters, Number of Switches, Max Cluster Size, Mean Cluster Size], [Age (in years)], [Maternal Education, Paternal Education].

## Data Availability

The Greek animal scheme file and the spelling file used for cluster analyses using the SNAFU Python library are openly available in OSF at https://doi.org/10.17605/OSF.IO/K6H3V. The children’s data presented in this study is available to interested parties on request.
